# Aspirin Exerts Neuroprotective Effects by Reversing Lipopolysaccharide-Induced Secondary Brain Injury and Inhibiting Matrix Metalloproteinase-3 Gene Expression

**DOI:** 10.1155/2021/3682034

**Published:** 2021-11-08

**Authors:** Depeng Feng, Dezhe Chen, Tuanzhi Chen, Xiaoqian Sun

**Affiliations:** Department of Neurology, Liaocheng People's Hospital and Liaocheng Clinical School of Shandong First Medical University, Shandong Province, China

## Abstract

**Objective:**

This study is aimed at exploring the possible neuroprotective mechanism of aspirin and the effect of bacterial endotoxin lipopolysaccharide (LPS) during cerebral ischaemia-reperfusion (CIRP) injury.

**Methods:**

We established three animal models: the CIRP, LPS, and CIRP+LPS models. Mortality, the injured brain area, and the beam walking test were used to estimate the degree of cerebral injury among the rats. Immunohistochemistry and immunofluorescence were used to detect activated microglia, matrix metalloproteinase-3 (MMP-3), and osteopontin (OPN).

**Results:**

The injured brain area and mortality were dramatically reduced (*p* < 0.01), and the beam walking test scores were elevated (*p* < 0.01) in the acetylsalicylic acid (ASA) group compared to the control group. The number of microglia-, MMP-3-, and OPN-positive cells also increased. Furthermore, the number of GSI-B4, OPN, and MMP-3 cells decreased in the ASA group compared to the control group. After LPS stimulation, the number of microglia reached a peak at 24 h; at 7 d, these cells disappeared. In the ASA group, the number of microglia was significantly smaller (*p* < 0.05), especially at 24 h (*p* < 0.01), compared to the LPS group. Moreover, the injured brain area and the mortality were dramatically increased and the beam walking test scores were reduced (*p* < 0.01) after LPS simulation following CIRP. The degree of injury in the ASA group resembled that in the control group. However, the number of MMP-3-immunoreactive neurons or microglia was significantly larger than that of the control group (*p* < 0.05). In the ASA group, the MMP-3 expression was also considerably decreased (*p* < 0.05).

**Conclusions:**

After CIRP, microglia were rapidly activated and the expression of MMP-3 and OPN significantly increased. For rats injected with LPS at reperfusion, the injured brain area and mortality also dramatically increased and the neurologic impairment worsened. However, ASA exhibited a neuroprotective effect during CIRP injury. Furthermore, ASA can reverse LPS-induced cerebral injury and inhibit the inflammatory reaction after CIRP injury.

## 1. Introduction

The pathophysiological mechanism of ischaemia reperfusion (I/R) injury is considerably complex, involving a series of damage cascades, such as oxidative stress, excitatory amino acid toxicity, Ca^2+^ overload, apoptotic gene expression, and inflammatory response [1]. The excessive inflammatory reaction of the brain tissue after cerebral I/R (CIRP) is one of the main causes of reperfusion injury and is also an important pathophysiological mechanism of secondary injury from cerebral ischaemia. The accumulation of inflammatory cells and the release of inflammatory factors at the early stage of I/R lead to the transition from ischaemic injury to inflammatory injury [2]. Acetylsalicylic acid (ASA), also known as aspirin, is a nonsteroidal anti-inflammatory drug with a wide range of pharmacological effects and multiple sites of action. In recent years, ASA has been found to have direct brain protection, in addition to antithrombotic effects in the treatment of ischaemic stroke [3, 4]. In this study, a rat middle cerebral artery occlusion model was used to analyse the effects of ASA on microglial activation and the expression of the matrix metalloproteinase-3 (MMP-3) and osteopontin (OPN) genes after CIRP and to explore the neuroprotective mechanisms of ASA.

## 2. Materials and Methods

### 2.1. Experimental Animal and Grouping

Healthy male Sprague–Dawley (SD) rats weighing between 240 g and 280 g from the Department of Medical Animals, General Hospital of Shenyang Military Region, China, were used in the three animal models of this study: the CIRP, lipopolysaccharide (LPS), and CIRP+LPS models. For the CIRP model, 66 SD rats were randomly divided into three groups: the sham group (*N* = 5) was given normal saline after surgery, the control group (*N* = 30) was given a solvent after CIRP, and the ASA group (*N* = 31) was given 80 mg/kg ASA after CIRP. Each group was further divided into five subgroups according to the time of execution: the 6 h, 24 h, 3 d, 5 d, and 7 d groups. For the PS model, 35 SD rats were randomly divided into three groups: the LPS group (*N* = 15) was intraperitoneally injected with 2.5 mg/kg LPS, the control group (*N* = 5) was treated with normal saline of the same volume, and the ASA group (*N* = 15) was intraperitoneally injected with 2.5 mg/kg LPS+80 mg/kg ASA. Each group was similarly divided into five subgroups according to time points: the 6 h, 24 h, 3 d, 5 d, and 7 d groups. For the CIRP+LPS model, the PS group (*N* = 11) was given 1 mg/kg LPS after CIRP and the LPS+ASA group (*N* = 10) was given 1 mg/kg LPS plus 80 mg/kg ASA after CIRP. Both groups were also divided into two subgroups according to time: the 24 h and the 7 d groups. After awakening from anaesthesia, the rats were returned to a cage where they could eat and drink freely. Rats from each group were sacrificed at the appropriate time point to obtain the necessary specimens. The study was approved by the Ethics Committee of Liaocheng People's Hospital.

### 2.2. Animal Model Preparation

For the CIRP model, the rats fasted 12 h before the operation, were anaesthetised with sodium pentobarbital (50 mg/kg) administered intraperitoneally, and then were operated on using a modified Zea Longa method [5]. Rats in the sham operation group were only treated with anaesthesia and blood vessel separation, and sutures were not introduced. Rats were screened for limb neurological deficit scores according to the Bederson 5 method, and those with a score of 1 to 3 were considered positive for CIRP. For the LPS model, the rats were intraperitoneally injected with 2.5 mg/kg of LPS [6]. For the CIRP+LPS model, during the CIRP model reperfusion, 1 mg/kg LPS was intraperitoneally administered to the rats immediately after the bolt was extracted.

### 2.3. Beam Walking Test

The recovery of sensory-motor coordination function was assessed using the beam walking test (BWT) by Ohlsson et al. [7, 8]. Training for 30 min per day 1 week before the operation was implemented, which enabled the rats to walk steadily on the crossbar. Four scores were then recorded 24 h, 3 d, 5 d, and 7 d after surgery.

### 2.4. Mortality Calculation

Deaths were used to calculate mortality during the observation period, which started 24 h after CIRP surgery.

### 2.5. Brain Injury Range Determination

Rats were anaesthetised with sodium pentobarbital (50 mg/kg) intraperitoneally administered at 24 h after I/R, and the brain was taken out after the rats were perfused with NS. Four to five coronal slices were obtained using a brain slicer (the first knife was at the midpoint of the front pole of the brain and the line of intersection, the second knife was at the optic chiasm, the third knife was at the handle of the funnel, and the fourth knife was between the funnel and the tail of the posterior leaf). The sections were incubated with 1% triphenyltetrazolium chloride solution in the dark at 37°C for 30 min and then soaked in 10% formalin. Image-Pro Plus 6.0 software was used to calculate the infarction area and total area of each slice, which were expressed as the percentage of the infarction area to the total area.

### 2.6. Immunofluorescence Staining

The brain samples for immunofluorescence staining were obtained as described in Section 2.5. After gradient elution with sucrose, the brain was subjected to routine dehydration, transparency, waxing, and embedding, among other steps, to obtain 5 *μ*m thick sections. The paraffin sections were dewaxed and repeatedly washed with 0.01 M PBS buffer. For the immunohistochemical staining of microglia lectin GSI-B4, biotinylated lectin GSI-B4 (diluted in 0.01 M PBS buffer at a final concentration of 5 *μ*g/mL) was added to the samples for 1 h at 37°C; then, the lectin was replaced with 0.01 M PBS buffer as a negative control. For the immunohistochemical staining of OPN and MMP-3, the appropriate dilution of the primary antibody (OPN antibody, 1 : 400; MMP-3, antibody 1 : 100) was added to the samples. These samples were incubated for 1 h at 37°C; then, the corresponding primary antibody was replaced with 0.01 M PBS buffer for the negative control. Subsequently, the corresponding biotin-labelled secondary antibody was added, and the samples were incubated at 37°C for 20 min. A fluorescence microscope was used for observation and to obtain photographs [9, 10]. The brain slices were analysed semiquantitatively using the ImageJ software.

### 2.7. Statistical Analysis

All data were expressed as mean ± standard deviation. One-way analysis of variance was used to analyse differences between multiple groups, and the *t*-test was used to analyse differences between two groups. Multiple comparisons of mean between samples were analysed using the SNK-q test. The chi-square test was used for comparisons of rat mortality and OPN-positive cell rates. Statistical significance was set at *p* < 0.05, and *p* < 0.01 was considered very significant. The SPSS software (version 20.0) was used for data analyses.

## 3. Results

### 3.1. Beam Walking Test

As shown in Table 1, the sham-operated group did not exhibit symptoms of neurological deficiency during all time points. The LPS group had the lowest score and the most severe neurological deficit, which was significantly different from the control group (*p* < 0.01). The level of neurological deficits in the ASA group was the lowest, which was likewise significantly different from the control group (*p* < 0.01). In addition, there was no significant difference in the degree of neurological recovery between the LPS+ASA group and the control group.

### 3.2. Effects of LPS and ASA on the Extent of Brain Injury

**Figure 1:**
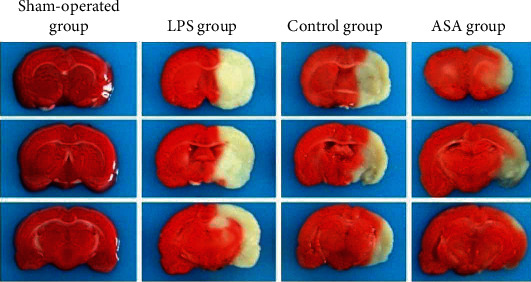
TTC-stained brain sections showing the effects of LPS and ASA on the extent of cerebral ischaemia/reperfusion brain injury. The white area represents the range of brain damage.

As shown in Table 2 and Figure 1, LPS significantly increased the extent of brain injury among treated rats compared to the control group (*p* < 0.01). The brain damage range of the ASA group was significantly smaller than that of the control group (*p* < 0.01). By contrast, the brain damage range of the LPS+ASA group was comparable to that of the control group but was significantly lower than that of the LPS group (*p* < 0.01).

### 3.3. The Impact of LPS and ASA on Mortality

As shown in Table 3, LPS significantly increased the mortality of rats during CIRP compared to the control group (*p* < 0.05). However, the mortality of the ASA group was significantly lower than that of the control group (*p* < 0.05), and that of the LPS+ASA group was similar to that of the control group.

### 3.4. Changes in Isolectin B4-Positive Cells across Different Time Points

**Figure 2:**
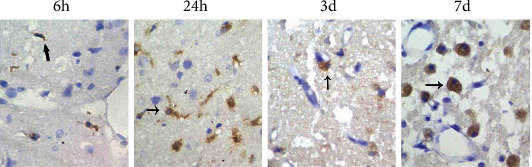
The immunohistochemical results of the control group: isolectin B4-positive cells at different time points after CIRP (×400).

Isolectin B4-positive cells were not detected in the nonischaemic brain tissue of sham-operated rats and CIRP rats. In the ischaemic infarction area, the morphology and number of microglia varied with the duration after CIRP and the ischaemic area location. As shown in Figure 2, only a small number of isolectin B4-positive cells were detected in the control group within the infarction centre 6 h after CIRP, and the observed cells were mainly rod-shaped and round cells. The number of microglia in the peri-infarction area reached a peak 24 h after CIRP, which was significantly different from the number at 6 h (*p* < 0.01), and most cells were round and high-branched. The number of round and amoebic microglia in the infarction centre increased further 3–5 d after CIRP. Furthermore, the number of microglia decreased in the infarction area but remained high. On the seventh day after CIRP, the maximum number of round or amoebic microglia was observed in the whole infarcted ischaemic area.

### 3.5. Effect of ASA on Isolectin B4-Positive Cells

As shown in Table 4, the number of microglia in the ASA treatment group was lower than that in the control group at each time point after CIRP. At 24 h, the number of microglia in the ASA group was significantly different from that in the control group (*p* < 0.01). Moreover, in the infarction core area, the number of microglia significantly decreased from 24 h to 7 d compared to the control group (*p* < 0.05).

### 3.6. Expression of OPN and MMP-3 and the Influence of ASA

**Figure 3:**
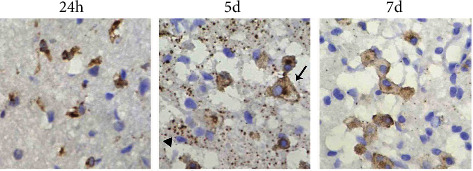
The immunohistochemical results of the control group: OPN-positive cells at different time points after CIRP (×400). OPN-positive small glial cells (arrow) and OPN protein particles (triangle) in the extracellular space.

OPN-positive cells were not labelled in the normal rats, the sham-operated groups, or the cerebral ischaemia group. OPN expression in OPN-positive cells was observed in the ischaemic infarction area from 6 h to 7 d after CIRP. The cell morphology and expression time course were consistent with that of isolectin B4-positive cells. As shown in Figure 3, OPN-positive cells in the control group were confined to the peri-infarction area 6 h after CIRP. Furthermore, we observed only a small number of cells, low immune response intensity, and light staining. At 24 h after CIRP, we observed that a large number of OPN-positive cells surrounded the infarction, and this was significantly different from that at 6 h. OPN-positive cells, mainly round and amoebic-like cells, also began to appear in the infarction zone. High-branched cells were also seen in the peripheral region. At 3 d after CIRP, the number of OPN-positive cells in the infarction centre significantly increased, and the cell body gradually increased as well. At 5 d, these cells became typical balloon-like cells: the cell body expanded to a balloon-like appearance, the nucleus and membrane were heavily stained, and the cytoplasm was lightly or not stained. At 5-7 d, a significant reduction of OPN-positive cells around the infarction, mainly amoebic-like and round cells, was observed. From the third day after CIRP, a large amount of OPN protein particles have been detected in the extracellular space, and by the seventh day, the OPN protein particles in the extracellular matrix became significantly reduced. The infarction area consisted mainly of balloon-like cells.

As shown in Tables 5 and 6, the number of OPN-positive cells in the ASA-administered group was slightly lower than that in the control group; however, this difference was not significant. Because isolectin B4-positive microglia was significantly lower in the ASA group than the control group, the positive rate of OPN microglia (positive rate = OPN positive cells/isolectin B4‐positive cells) in the ASA group was significantly higher than that in the control group (*p* < 0.01).

**Figure 4:**
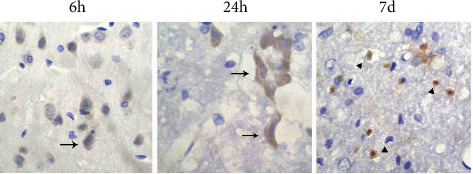
The immunohistochemical results of the control group: MMP-3-positive cells at different time points after CIRP (×400).

With respect to MMP-3 expression and the effects of ASA, no MMP-3-positive cells were detected in the brain tissue of normal and sham-operated rats. MMP-3 was more widely expressed in the ischaemic and nonischaemic neurons 6 h to 7 d after CIRP, especially in the cerebral cortex. As shown in Figure 4, the control group showed a large number of MMP-3-positive cells in the peri-infarction and central areas 6 h after CIRP and became most obvious at 24 h to 3 d. The cytoplasm and nucleus of these cells were condensed and had a triangular appearance, which is consistent with that of a typical ischaemic neuron. From 3 d after CIRP, a greater number of round and amoebic MMP-3-positive microglia appeared in the peri-infarct area. These cells reached a maximum number and also became expressed in the infarction centre at 7 d. In addition, MMP-3 was expressed in perivascular cells and basal membranes from 24 h to 3 d after CIRP. MMP-3 was also expressed in the hippocampus and ventricular choroid plexus of the ischaemic side at 5 d to 7 d.

As shown in Table 7, compared to the control group, the MMP-3-positive ischaemic neurons in the infarcted area of the ASA treatment group were significantly reduced, with the most significant reduction at 3–7 d (*p* < 0.05).

### 3.7. Activation of Microglia in the LPS Model Group and the Effects of ASA

**Figure 5:**
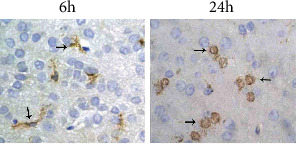
The immunohistochemical results of isolectin B4-positive cells in the LPS group at different time points after LPS injection (×100).

Isolectin B4-positive microglia were not detected in the brain tissue of the rats in the control group. As shown in Figure 5, the immunostaining of microglia in the LPS group became apparent 6 h after LPS administration. Their morphology was high-branched, the synapse was slender, and they were not fully activated. The number of cells did not significantly change at 24 h; nevertheless, their morphology changed. The protrusions became retracted, forming circular activated microglia, and isolectin B4 staining became strongly positive. On the third day, the morphology of the microglia was the same as that at 6 h, which was highly branched. After 5 d, the number of microglia significantly decreased. On the seventh day, isolectin B4-positive microglia disappeared.

As shown in Table 8, the number of isolectin B4-positive cells in the ASA group was significantly lower than that in the LPS group (*p* < 0.05) at 24 h (*p* < 0.01). This duration was short, and activation was observed on the fifth day after LPS injection. The small glial cells have almost disappeared, which was earlier than that in the LPS group (disappeared on the seventh day).

### 3.8. Expression of Microglia and MMP-3 in the CIRP+LPS Model Group and the Effect of ASA

**Figure 6:**
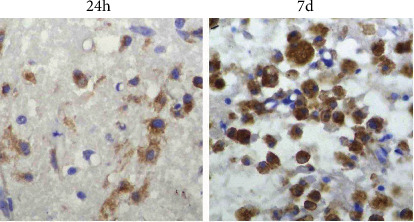
The immunohistochemical results of isolectin B4-positive cells in the LPS group of the CIRP+LPS model at different time points (×400).

After intraperitoneal injection of LPS, the number of microglia and the intensity of immune response in the rats became significantly different than that in the control group, as well as when compared to the surrounding area. As shown in Figure 6, a large number of round and amoebic cells were seen in the infarction core 24 h after CIRP, with foam cells also evident. A large number of amoebic and highly branched cells were seen in the area around the infarction. At 7 d, foam cells became distributed throughout the entire ischaemic infarction area, and the number of these cells was significantly different from that in the control group (*p* < 0.01).

As seen in Table 9, the number of microglia in the ASA treatment group was significantly lower than that in the LPS group, and their number and morphology were comparable to those in the control group.

**Figure 7:**
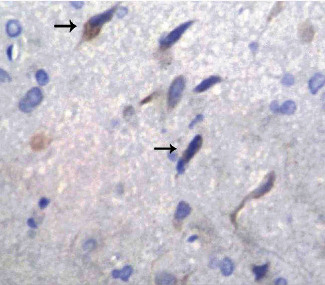
MMP-3-positive cells in the CIRP+LPS group at 7 d (×400).

After intraperitoneal injection of LPS, the area around the cerebral infarction enlarged and the number of ischaemic neurons significantly increased. The corresponding MMP-3-positive neurons also became significantly increased compared to the control group, and the morphological changes of MMP-3-positive ischaemic neurons became more apparent. The cell body became extremely condensed and a thin rod shape became evident (Figure 7).

As seen in Table 10, MMP-3 expression was significantly lower in the ASA-treated group than in the LPS group but was similar to the control group.

## 4. Discussion

Microglia are widely distributed in the central nervous system and are macrophages inherent in the brain. A few hours after cerebral ischaemia, the inflammatory reaction in the damaged brain region begins for several days, which aggravates the delayed brain injury caused by cerebral ischaemia and worsens the biological function of nerve cells [11]. Glial cells are important in the immune-inflammatory response; therefore, the role of microglia in cerebral ischaemic injury has been receiving increasing attention [12]. In our experiment, a model of transient cerebral artery occlusion was established by the suture method. Microglia activation after CIRP was observed through immunohistochemistry. After CIRP, the morphology and number of microglia were observed to have an association with time and the ischaemic area. Isolectin B4 was used to label five different forms of activated microglia, including inactivated, highly branched rod-shaped, fully activated round and amoebic cells, and terminally phagocytic necrotic tissue cell foam cells. In the peri-infarction area, a large number of microglia appeared 6 h after CIRP, and these cells were banded around the infarction centre. The number of activated microglia reached a peak at 24 h, and its range expanded. Semiactivated high-branched microglia also began to appear in the ischaemic periphery and the normal area of the brain tissue. The number of microglia in the peri-infarction area decreased at 3–7 d but remained at a high level. Furthermore, its activation increased, and most were round and amoebic. The appearance time and peak time of microglia in the central part of the infarction were significantly later than that in the infarction area, and by the seventh day, these cells filled the necrotic area. Studies have found that microglia participate in the process of ischaemic neuron damage at the early stage of ischaemia and that they may play an important role in promoting repair during the late stages of brain injury [13]. Our experiment demonstrated the inhibitory effect of ASA on microglia activation in three aspects. First, after CIRP, ASA significantly inhibited the number and affected the morphology of isolectin B4-positive microglia in the ischaemic penumbra or infarction centre. Second, ASA exhibited a significant inhibitory effect on the activation of LPS-induced microglia. Third, ASA reversed the activation of microglia induced by CIRP injury and LPS dual factors in the control group. In contrast to the neuroprotective mechanism of ASA and the mechanism of microglial activation, it can be inferred that ASA inhibited the activation of microglia through at least two pathways. The first is the inhibition of the key transcription factor NF-*κ*B and the inflammatory factors TNF-*α* and IL-1*β* in an acute inflammatory response. The second is by reducing the activation of ischaemic neurons, especially the generation of neuronal apoptosis, reducing the signalling from injured neurons, and thereby inhibiting the activation of microglia. Therefore, ASA could have numerous inhibitory effects on microglial activation. ASA may act as a regulator to alleviate the malignant circulation of the inflammatory cascade centred on the microglia after CIRP and exert neuroprotective effects.

MMPs are a group of zinc-dependent proteolytic enzymes that function to degrade a variety of extracellular matrix components. Under pathological conditions, MMPs can promote the development of many diseases, such as tumor invasion and infiltration, atherosclerosis, multiple sclerosis, Alzheimer's disease, malignant glioma, and other central nervous system diseases [14, 15]. In this study, the immunohistochemical method was used to observe MMP-3 expression after CIRP. We found that MMP-3 was mainly expressed in neurons and microglia in the ischaemic infarction area, similar to previous findings [16]. In the central part of the infarction, MMP-3-positive neurons mainly appeared before 24 h and disappeared after 3 d, which was related to the necrosis and disintegration of most of the neurons in the infarction area. In the peri-infarction area, MMP-3-positive neurons exhibited a strong immune response, a large number, and a long course, reaching a peak on the third day and continuing until the seventh day. We observed that MMP-3 can be expressed both in the neurons of the ischaemic necrosis and in the neurons with delayed apoptosis onset. Reportedly, MMP-3 positive microglia mainly exist in the infarction centre 4–7 d after reperfusion in a rat cerebral ischaemia 50 min reperfusion model [17]. Furthermore, it was also found that MMP-3 is also expressed in the perivascular cells, basement membrane, and ventricular choroid plexus in the infarction area, which may be involved in the degradation of the vascular basement membrane after cerebral ischaemia, destroying the BBB, and leading to leakage of plasma protein into the brain parenchyma or to brain oedema. Recent studies have also found that ASA is a nonspecific inhibitor of MMPs. ASA can inhibit cell MMP-2 activity in different types of diseases [18–20]. In the present study, we observed that ASA can reduce the role of MMP-3-positive ischaemic neurons in the peri-infarction zone after CIRP in rats and that ASA can also reverse LPS induction as well as high MMP-3 expression. The inhibitory effect of ASA on MMP-3 expression after cerebral ischaemia may be due to the following reasons. First, ASA directly inhibited the expression of MMP-3 by some means, similar to other MMPs. Second, ASA inhibited neuronal apoptosis after CIRP, such that the number of ischaemic neurons decreased, and the number of MMP-3-positive neurons became correspondingly reduced. However, the inhibitory effect of ASA on MMP-3 expression after cerebral ischaemia needs further investigation. Our findings suggest that ASA inhibits MMP-3 at the early stage of cerebral ischaemia. On the one hand, it alleviates the damaging effect of MMPs on BBB, and on the other hand, it attenuates the activation of MMP-3 on microglia, thereby exerting neuroprotective effects.

OPN is a component of the extracellular matrix of normal central nervous tissue and a key factor in tissue repair and extracellular matrix remodeling after central nervous system injury. In this study, OPN expression after CIRP was evaluated through immunohistochemistry, and we found that OPN was mainly expressed in the microglia. Expression was evident mainly in the peri-infarction area at 6 h to 3 d and peaked at 24 h. After 5–7 d, it was present in the infarction as well. In the core region, large amounts of OPN protein particles were secreted into the extracellular matrix starting from the third day. In vitro experiments have shown that OPN can activate astrocytes and promote the migration process of astrocytes by binding to the integrin receptor *α*V*β*3 [21]. These results indicate that after ischaemic brain injury, OPN and its receptors promote the activation, migration, and scar formation of glial cells after local cerebral infarction and participate in the tissue repair process. In our study, we observed that the number of OPN-positive cells decreased after ASA administration compared to the control group. OPN expression after CIRP was mainly observed in the microglia, and ASA has a significant inhibitory effect on the microglia. Therefore, these factors could be related to the observed decrease in the number of OPN cells. Studies have shown that OPN can play a protective role against ischaemic injury through multiple intracellular pathways and downstream effector proteins, including decreased iNOS expression, activation of protein kinase Akt, increased expression of antiapoptotic protein Bcl-2, and activation of the phosphatidylinositol 3-kinase (PI3-K)/Akt and P42/44 MAPK pathways [22].

LPS, a bacterial endotoxin, is often used as a polyclonal immune stimulator in immune response studies to mimic the immune status of the body. It is a common model for studying the information exchange between the immune system and the nervous system [23]. Our study investigated the plasticity changes of microglia after a single intraperitoneal injection of LPS in normal rats and CIRP rats. Microglia were activated several hours after intraperitoneal injection of LPS in normal rats, with the highest number from 6 h to 3 d, lasting until the seventh day after the injection. At 24 h, the microglia exhibited a round appearance and was fully activated, indicating that 2.5 mg/kg of LPS can cause morphological changes. After the intraperitoneal injection of CIRP rats with LPS, t microglial activation became more significant; the number of microglia not only increased but morphologically large macrophage-like cells were also observed. In vitro experiments revealed that LPS did not exert direct toxic damage to neurons. The effect of LPS on the central nervous system is triggered by microglial activation. Activated microglia can produce inflammatory or cytotoxic factors such as TNF-*α*, IL-*β*, COX-2, iNOS, and superoxide, which can damage surrounding neurons [24, 25]. Therefore, this process is often used to reverse microglial activation caused by LPS as an objective indicator for evaluating neuroprotection [26, 27]. In our experiment, LPS was used to simulate the inflammatory process in the brain after systemic infection. The results suggest that LPS aggravates microglial activation and MMP-3 expression after CIRP, which aggravates the inflammatory cascade after cerebral ischaemia, thereby leading to secondary inflammatory brain injury and aggravating neurological deficit after acute stroke. The results of this experiment also revealed that an intraperitoneal ASA injection after CIRP exerted significant neuroprotective effects and suggested that ASA was at least partially neuroprotective by inhibiting the inflammatory response after CIRP injury.

In conclusion, this experiment used some animal models from several aspects to study microglial activation, MMP-3 and OPN expression, and the effect of ASA on these processes. Owing to the limitations of applied animal in vivo research, immunohistochemistry is only used for semiquantitative studies. The mechanism of action of ASA observed in our experiment should therefore be confirmed by in vitro experiments, such as cell culture methods from the cellular and molecular levels of research. Moreover, further discussion on the neuroprotective mechanism of ASA can provide not only clinical treatment but also a fundamental theoretical basis for the modification of ASA, that is, the construction of the so-called super aspirin.

## Figures and Tables

**Table 1:** Beam walking test scores of each group ($\overset{¯}{x}\pm s$).

Group	Scores at each time point			
	24 h	3 d	5 d	7 d
Sham-operated group	5.80 ± 0.40	5.85 ± 0.35	6.00 ± 0.00	6.00 ± 0.00
Control group	1.75 ± 0.58	2.67 ± 0.51	3.45 ± 0.62	4.31 ± 0.46
ASA group	2.35 ± 0.56^∗^	3.44 ± 0.44^∗^	4.20 ± 0.41^∗^	5.10 ± 0.50^∗^
LPS group	1.10 ± 0.41^∗^	1.52 ± 0.50^∗^	2.73 ± 0.60^∗^	3.50 ± 0.55^∗^
LPS+ASA group	1.70 ± 0.63^#^	2.35 ± 0.49^#^	3.35 ± 0.36^#^	4.11 ± 0.47^#^

^∗^ *p* < 0.01 compared to the control group; ^#^*p* < 0.01 compared to the LPS group.

**Table 2:** The influence of LPS and ASA on the range brain injury 24 h after cerebral ischaemia/reperfusion ($\overset{¯}{x}\pm s$).

Group	Number of specimens (*n*)	Brain damage range (%)
Control group	5	26.40 ± 1.51
ASA group	6	11.08 ± 0.44^∗^
LPS group	5	35.80 ± 2.50^∗^
LPS+ASA group	4	23.35 ± 1.49^#^

^∗^ *p* < 0.01 compared to the control group; ^#^*p* < 0.01 compared to the LPS group.

**Table 3:** The effects of LPS and ASA on the mortality of rats 24 h after cerebral ischaemia/reperfusion.

Group	Number of surgical animals	Number of deaths	Mortality (%)
Sham-operated group	5	0	0
Control group	31	6	19.3
ASA group	29	3	10.3^∗^
LPS group	15	5	33.3^∗^
LPS+ASA group	15	3	20.0

^∗^ *p* < 0.05 compared to the control group.

**Table 4:** Number of isolectin B4-positive cells after CIRP/10 × 40 field of view.

Time points	Core area of infarction		Peri-infarction area	
	Control group	ASA group	Control group	ASA group
6 h	3.1 ± 1.5	1.2 ± 0.5	24.4 ± 5.3	15.2 ± 3.9^∗^
24 h	22.4 ± 6.5	10.9 ± 5.7^∗^	45.1 ± 8.1	18.5 ± 5.7^∗∗^
3 d	29.5 ± 7.0	13.4 ± 4.6^∗^	33.5 ± 7.5	25.4 ± 4.8^∗^
5 d	40.4 ± 6.8	25.8 ± 6.2^∗^	31.7 ± 9.1	24.1 ± 7.3^∗^
7 d	48.7 ± 8.4	33.4 ± 7.6^∗^	30.6 ± 6.7	22.3 ± 5.6^∗^

^∗^ *p* < 0.05 and ^∗∗^*p* < 0.01 compared to the control group.

**Table 5:** The effect of ASA on the rate of OPN-positive cells in the peri-infarction area after CIRP.

	24 h		3 d	
	Control group	ASA group	Control group	ASA group
Isolectin (+)	45.1 ± 8.1	18.5 ± 5.7	33.5 ± 7.5	25.4 ± 4.8
OPN (+)	23.5 ± 6.7	16.2 ± 5.4	16.3 ± 6.0	21.0 ± 5.2
Positive rate	52.1%	87.6%^∗^	48.7%	66.9%^∗^

^∗^ *p* < 0.01 compared to the control group.

**Table 6:** The effect of ASA on the rate of OPN-positive cells in the core infarction area after CIRP.

	5 d		7 d	
	Control group	ASA group	Control group	ASA group
Isolectin (+)	40.4 ± 6.8	25.8 ± 6.2	48.7 ± 8.4	33.4 ± 7.6
OPN (+)	25.5 ± 7.3	20.5 ± 7.4	31.3 ± 8.0	24.9 ± 8.1
Positive rate	63.1%	79.4%^∗^	64.3%	74.6%^∗^

^∗^ *p* < 0.05 compared to the control group.

**Table 7:** The effect of ASA on the number of MMP-3-positive ischaemic neurons in the peri-infarction area.

	6 h	24 h	3 d	5 d	7 d
Control group	11.5 ± 4.8	16.8 ± 6.2	23.8 ± 6.9	21.7 ± 5.4	17.4 ± 3.6
ASA group	9.5 ± 3.3	12.5 ± 4.3	16.5 ± 4.5^∗^	13.3 ± 3.0^∗^	10.4 ± 2.1^∗^

^∗^ *p* < 0.05 compared to the control group.

**Table 8:** Number of isolectin B4-positive cells at different time points after intraperitoneal injection of LPS.

	6 h	24 h	3 d	5 d	7 d
LPS group	35.6 ± 5.0	40.5 ± 5.6	30.3 ± 6.2	15.2 ± 2.9	3.1 ± 2.0
ASA group	21.1 ± 3.8^∗^	23.1 ± 4.6^∗∗^	16.5 ± 5.4^∗^	2.8 ± 1.3^∗^	0.0 ± 0.0

^∗^ *p* < 0.05 and ^∗∗^*p* < 0.01 compared to the control group.

**Table 9:** Number of isolectin B4-positive cells across different groups/10 × 40 field of view.

Group	CIRP after 24 h		CIRP after 7 d	
	Core area	Surrounding area	Core area	Surrounding area
Control group	22.4 ± 6.5	45.1 ± 8.1	48.7 ± 8.4	30.6 ± 6.7
ASA group	10.9 ± 5.7^∗^	18.5 ± 5.7^∗^	33.4 ± 7.6^∗^	22.3 ± 5.6^∗^
LPS group	35.1 ± 7.0^∗^	59.4 ± 8.6^∗^	85.0 ± 17.5^∗∗^	52.4 ± 4.8^∗∗^
LPS+ASA group	25.3 ± 5.8^#^	42.8 ± 7.2^#^	53.±14.1^##^	33.1 ± 8.3^##^

^∗^ *p* < 0.05 and ^∗∗^*p* < 0.01 compared to the control group; ^#^*p* < 0.05 and ^##^*p* < 0.01 compared to the LPS group.

**Table 10:** The number of MMP-3-positive ischaemic neurons across different groups in the peri-infarction area.

	24 h	7 d
Control group	16.8 ± 6.2	17.4 ± 3.6
ASA group	12.5 ± 4.3	10.4 ± 2.1^∗^
LPS group	30.3 ± 7.5^∗^	25.3 ± 5.3^∗^
LPS+ASA group	15.1 ± 5.4^#^	13.3 ± 6.7^#^

^∗^ *p* < 0.05 compared to the control group; ^#^*p* < 0.05 compared to the LPS group.

## Data Availability

The data used to support the findings of this study are available from the corresponding author upon request.

## References

[1] Ding X.-S., Feng M.-J. (2006). Pathophysiology and apoptosis in ischemic stroke. *International Journal of Cerebrovascular Diseases*.

[2] Gill M. B., Bockhorst K., Narayana P., Perez-Polo J. R. (2008). Bax shuttling after neonatal hypoxia-ischemia: hyperoxia effects. *Journal of Neuroscience Research*.

[3] Fu Y., Zhen J., Lu Z. (2017). Synergetic neuroprotective effect of docosahexaenoic acid and aspirin in SH-Y5Y by inhibiting miR-21 and activating RXR*α* and PPAR*α*. *DNA and Cell Biology*.

[4] Parmar H. S., Houdek Z., Pesta M., Vaclava C., Dvorak P., Hatina J. (2017). Protective effect of aspirin against oligomeric A*β*42 induced mitochondrial alterations and neurotoxicity in differentiated EC P19 neuronal cells. *Current Alzheimer Research*.

[5] Longa E. Z., Weinstein P. R., Carlson S., Cummins R. (1989). Reversible middle cerebral artery occlusion without craniectomy in rats. *Stroke*.

[6] Choi J. S., Park H. J., Cha J. H., Chung J. W., Chun M. H., Lee M. Y. (2003). Induction and temporal changes of osteopontin mRNA and protein in the brain following systemic lipopolysaccharide injection. *Journal of Neuroimmunology*.

[7] Silveira E. M. S., Kroth A., Santos M. C. Q. (2019). Age-related changes and effects of regular low-intensity exercise on gait, balance, and oxidative biomarkers in the spinal cord of Wistar rats. *Brazilian Journal of Medical and Biological Research*.

[8] Fréchou M., Margaill I., Marchand-Leroux C., Beray-Berthat V. (2019). Behavioral tests that reveal long-term deficits after permanent focal cerebral ischemia in mouse. *Behavioural Brain Research*.

[9] Du X., Wang X., Cui K. (2021). Tanshinone IIA and astragaloside IV inhibit miR-223/JAK2/STAT1 signalling pathway to alleviate lipopolysaccharide-induced damage in nucleus pulposus cells. *Disease Markers*.

[10] Xu Y., Wang Q., Wu Z. (2019). The effect of lithium chloride on the attenuation of cognitive impairment in experimental hypoglycemic rats. *Brain Research Bulletin*.

[11] Eldahshan W., Fagan S. C., Ergul A. (2019). Inflammation within the neurovascular unit: focus on microglia for stroke injury and recovery. *Pharmacological Research*.

[12] Kata D., Földesi I., Feher L. Z., Hackler L., Puskas L. G., Gulya K. (2017). A novel pleiotropic effect of aspirin: beneficial regulation of pro- and anti-inflammatory mechanisms in microglial cells. *Brain Research Bulletin*.

[13] Neumann J., Gunzer M., Gutzeit H. O., Ullrich O., Reymann K. G., Dinkel K. (2006). Microglia provide neuroprotection after ischemia. *The FASEB Journal*.

[14] Tokuhara C. K., Santesso M. R., Oliveira G. S. N. (2019). Updating the role of matrix metalloproteinases in mineralized tissue and related diseases. *Journal of Applied Oral Science*.

[15] Juurikka K., Butler G. S., Salo T., Nyberg P., Åström P. (2019). The role of MMP8 in cancer: a systematic review. *International Journal of Molecular Sciences*.

[16] Rosenberg G. A., Cunningham L. A., Wallace J. (2001). Immunohistochemistry of matrix metalloproteinases in reperfusion injury to rat brain: activation of MMP-9 linked to stromelysin-1 and microglia in cell cultures. *Brain Research*.

[17] Solé S., Petegnief V., Gorina R., Chamorro Á., Planas A. M. (2004). Activation of matrix metalloproteinase-3 and agrin cleavage in cerebral ischemia/reperfusion. *Journal of Neuropathology and Experimental Neurology*.

[18] Kuliczkowski W., Radomski M., Gąsior M. (2017). MMP-2, MMP-9, and TIMP-4 and response to aspirin in diabetic and nondiabetic patients with stable coronary artery disease: a pilot study. *Biomed Res Int*.

[19] Nicolae M., Tircol M., Alexandru D. (2005). Inhibitory effect of acetylsalicylic acid on matrix metalloproteinase -2 activity in human endothelial cells exposed to high glucose. *Journal of Cellular and Molecular Medicine*.

[20] Bhatt L. K., Veeranjaneyulu A. (2014). Enhancement of matrix metalloproteinase 2 and 9 inhibitory action of minocycline by aspirin: an approach to attenuate outcome of acute myocardial infarction in diabetes. *Archives of Medical Research*.

[21] Wang X., Louden C., Yue T. L. (1998). Delayed expression of osteopontin after focal stroke in the rat. *The Journal of Neuroscience*.

[22] Gary D. S., Milhavet O., Camandola S., Mattson M. P. (2003). Essential role for integrin linked kinase in Akt-mediated integrin survival signaling in hippocampal neurons. *Journal of Neurochemistry*.

[23] Batista C. R. A., Gomes G. F., Candelario-Jalil E., Fiebich B. L., de Oliveira A. C. P. (2019). Lipopolysaccharide-induced neuroinflammation as a bridge to understand neurodegeneration. *International Journal of Molecular Sciences*.

[24] Molina-Gonzalez I., Miron V. E. (2019). Astrocytes in myelination and remyelination. *Neuroscience Letters*.

[25] Godbout J. P., Berg B. M., Kelley K. W., Johnson R. W. (2004). *α*-Tocopherol reduces lipopolysaccharide-induced peroxide radical formation and interleukin-6 secretion in primary murine microglia and in brain. *Journal of Neuroimmunology*.

[26] Lei B., Mace B., Dawson H. N., Warner D. S., Laskowitz D. T., James M. L. (2014). Anti-inflammatory effects of progesterone in lipopolysaccharide-stimulated BV-2 microglia. *PLoS One*.

[27] Tweedie D., Frankola K. A., Luo W., Li Y., Greig N. H. (2011). Thalidomide analogues suppress lipopolysaccharide-induced synthesis of TNF-*α* and nitrite, an intermediate of nitric oxide, in a cellular model of inflammation. *The Open Biochemistry Journal*.

